# A Langasite Crystal Microbalance Coated with Graphene Oxide-Platinum Nanocomposite as a Volatile Organic Compound Sensor: Detection and Discrimination Characteristics

**DOI:** 10.3390/s20020334

**Published:** 2020-01-07

**Authors:** Ainan Leong, Tridib Saha, Varghese Swamy, Narayanan Ramakrishnan

**Affiliations:** 1 Discipline of Electrical and Computer Systems Engineering, School of Engineering, Monash University Malaysia, Bandar Sunway 47500, Selangor, Malaysia; ainan.leong@monash.edu (A.L.); tridib.saha@monash.edu (T.S.); 2 Discipline of Mechanical Engineering, School of Engineering, Monash University Malaysia, Bandar Sunway 47500, Selangor, Malaysia; varghese.swamy@monash.edu

**Keywords:** VOCs, gas sensor, acoustic sensor, graphene oxide, thickness shear mode resonator

## Abstract

We propose a novel langasite crystal microbalance (LCM) sensor with a graphene-based sensing medium to detect and discriminate volatile organic compounds (VOCs) at room temperature. A thin film of graphene oxide embedded with Pt nanostructures (GO-Pt nanocomposite) was deposited on the electrode surface of the LCM, a thickness-shear acoustic wave resonator. Ethyl acetate, acetic acid, and ethanol were chosen as typical VOCs for this study. Sensitivity and selectivity of coated LCM were investigated for different concentrations of the VOCs by analysing the resonant properties of the sensor. When exposed to VOCs, a negative shift in series resonance frequency was observed due to the mass loading of VOC molecules. Simultaneously, changes in equivalent resistance and parallel resonance frequency of the sensor were also observed due to the interaction of VOCs with charge carriers on the GO-Pt nanocomposite film surface. This dual measurement of both series and parallel resonance frequencies allowed for detection and discrimination of VOCs. Moreover, the high thermal stability of langasite makes the proposed sensor suitable even for harsh environmental conditions.

## 1. Introduction

Volatile organic compounds (VOCs) are encountered in every aspect of our daily life. Some VOC molecules are non-toxic, while others are detrimental to our wellbeing in terms of health and safety. Hence, it is of paramount importance to be able to sense these VOC molecules when present in the environment. The sensing of VOCs plays a critical role in various fields such as medical diagnostics, food quality control, environmental surveillance, and explosives detection [1,2]. It is simply insufficient to be able to sense the VOC molecules; it is even more significant to distinguish the VOC molecule types as they have similar physical and chemical properties.

Various methods have been adopted to achieve selectivity in a gas sensor [3,4,5,6]. A sensor array in which each sensor is coated with a distinct sensing material has been demonstrated for gas selectivity [3]. The sensors in the array would respond with the highest sensitivity towards specific analyte gases depending on the affinity of the individual sensing medium employed for selectivity to gases. Moreover, a multivariable sensor with several independent response parameters has the advantages of rejecting interferences as well as differentiating the analyte gases [7]. Each of the independent response variables tracks the distinct perturbations in the characteristics of the sensing layer including the physical, electrical, and chemical properties. By statistically analyzing the individual responses of the sensor array and the independent response variables of the multivariable transducer, the information on gas selectivity can be unraveled [3,8].

In general, a VOC sensor comprises an active sensing material and a transducer device to output the sensing signal that reflects the interactions between the sensing material and the gas molecules. Commercial VOC sensors available currently are predominantly based on metal oxide sensing layer which necessitates a heating element to operate [1]. This limits the uses of the metal oxide-based sensors to static scenarios and are, therefore, inappropriate for modern applications such as wearables where portability, low power consumption, and ambient temperature sensing are essential [7]. Furthermore, these commercial sensors merely sense the total VOCs present and lack the ability to discriminate the type of VOC molecules. Hence, there is a surge in demand for selective room temperature (RT) sensing of VOC molecules for daily air quality and health monitoring by consumers [7].

The contemporary increase in the popularity of two-dimensional (2D) materials has led to extensive research and development of selective detection of VOC molecules by applying the former as the sensing material [2]. The tremendous attention given to 2D materials, particularly graphene and its derivatives, is attributed to their fascinating yet peculiar properties [9]. Among the exceptional characteristics the most relevant property to sensing VOC is graphene’s remarkable specific surface area (2600 m^2^/g) which presents an extensive site for adsorption of molecules [10]. However, graphene in its pristine state has inferior molecular sensing performance due to its relatively inert surface [11]. Decoration of pristine graphene with noble metal nanoparticles, organic functional groups, and heteroatoms has been shown to improve the sensing performance of graphene-based VOC sensors [12,13]. Graphene oxide (GO), graphene with attached organic functional groups, presents an enormous prospect for enhanced VOC sensing performance owing to its tunable semiconductive character in contrast to the intrinsic conductive nature of pristine graphene [14,15]. Deposition of precious metal nanoparticles including silver, gold, and platinum on GO surface has been reported to promote RT molecular sensing by catalyzing the interactions between the adsorption sites and the target molecules [12,16,17]. Thus, GO in combination with noble metal nanoparticles offers an excellent sensing material for molecular detection.

It is essential to note that most of the graphene-based gas sensors reported in the literature have utilized sensor architectures in the form of a transistor or chemiresistor [8,12,16,17]. Gas sensors based on graphene in combination with a piezoelectric microbalance, are however, scarcely reported. Quartz crystal microbalance (QCM), a form of piezoelectric microbalance has been employed as a gas sensor in some work, but the sensing performance of such devices has the potential to be further improved [18,19]. The improvements may include employing the relatively new LCM, instead of QCM, that promises better sensitivity due to higher electromechanical coupling coefficient and piezoelectric stability from ambient to high temperatures, up to 1200 °C [20]. LCM has already been demonstrated as a viable VOC sensor [21]. However, no information on its gas selectivity has been reported. To the best of our knowledge, there is no report to date that integrates GO and LCM to detect and discriminate VOC molecules. In this paper we report the first implementation of a VOC sensor that combines LCM with Pt nanoparticle-decorated GO as the sensing layer for selective VOC molecular detection at RT. A comprehensive characterization of the sensing material was performed with the use of field-effect scanning electron microscopy (FESEM), Raman spectroscopy, and X-ray photoelectron spectroscopy (XPS) which indicated successful deposition of GO-Pt nanocomposite on the LCM sensing surface. The as-fabricated sensor exhibited good sensitivity and selectivity towards VOC molecules from different families such as acetic acid, ethanol, and ethyl acetate. Furthermore, the sensor characteristics were explained based on multiple output parameters and equivalent circuit of the LCM.

## 2. Materials and Methods

### 2.1. Sample Preparation

An LCM (Fomos-Materials, Moscow, Russia) piezoelectric resonator operating at a resonance frequency of 6 MHz was employed as the sensor device. The LCM has a pair of asymmetric electrodes: a 3 mm the top electrode for sensing and a 14 mm bottom electrode serving as the reference. This asymmetrical electrode structure enables the LCM to measure the electrical perturbation in addition to the common mass loading detection of a thickness shear mode (TSM) device. Commercially available GO solution produced through Hummers’ method (GO Advanced Solutions, Kuala Lumpur, Malaysia) was spin-coated onto the sensing electrode of the LCM. The spin-coating parameters include a maximum speed of 2000 rpm with an acceleration of 1000 revolution/s^2^ for a total spin duration of 18 s. The mass loading of the GO sensing film introduced resonance frequency shift of—916 Hz. Subsequently, nanoscale Pt was added to the GO-coated LCM (GO-LCM) using a rotary pump coater (Quorom Technologies, Lewes, UK) under low vacuum (1.0 × 10^−2^ mbar) for 60 sec. The Pt-embedded GO-LCM (Pt-GO-LCM) was then annealed under high vacuum (2.5 × 10^−6^ mbar) at 300 °C for 1 h inside a steel vacuum chamber to prevent the agglomeration of the Pt nanostructures. It is important to note that there are no high temperature phase transition(s) in langasite crystal structure and, therefore, its piezoelectric properties are unaltered to very high temperatures (1200 °C). This is in contrast to the behavior of a typical QCM wherein the piezoelectric properties of quartz start to degrade above 200 °C due to minor structural changes leading to a phase transition.

### 2.2. Gas Sensing Setup

**Figure 1:**
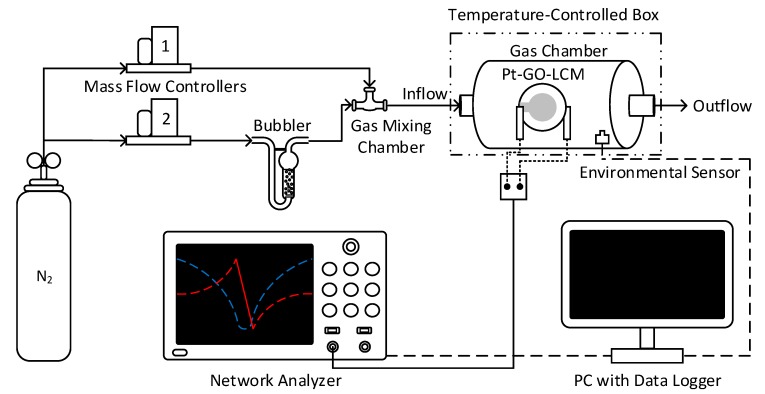
The automated gas sensing experimental setup.

The gas sensing experimental setup used (Figure 1) is equipped with two mass flow controllers, designated MFC1 and MFC2. Within the gas chamber (Donewell Resources, Kuala Lumpur, Malaysia) a metallic holder is used to secure the Pt-GO-LCM VOC sensor. A commercial BME680 environmental sensor (Bosch Sensortec, Gerlingen, Germany) is mounted inside the gas chamber to continuously monitor the conditions including temperature, relative humidity (RH), and absolute pressure. The serial port of a PC was connected to the BME680 sensor to log the test conditions. The gas chamber has a fixed volume of approximately 210 cm^3^ with two peripheral holes as the inlet and outlet. The inlet and outlet are always open for the continuous flow of gas mixture through the chamber to ensure a constant gas pressure inside the gas cell. Purified nitrogen gas supplied from a gas tank (Alpha Gas Solution, Kuala Lumpur, Malaysia) at a pressure of 1.5 bar is regulated by both MFCs (Hitachi Metals, Tokyo, Japan). Initially, the N_2_ regulated by MFC1 at 550 sccm is flowed through the gas chamber to establish a baseline sensor response and test environment conditions. The total flowrate, *V_total_*, of gas flowing through the gas chamber is maintained at 550 sccm throughout the experiment. To produce VOCs vapors at various concentrations, the flow of N_2_ carrier gas is controlled by MFC2 at three fixed flow rates of 15, 30 and 60 sccm. The carrier gas with flow rate, *V_carrier_*, is flowed through the bubbler to generate VOC vapors. The concentrated VOC vapors are diluted to a desired concentration by mixing with N_2_ introduced at a flow rate, *V_dilution_* and a specific flow ratio, *V_carrier_*/*V_total_.* The *V_total_* is defined as the sum of *V_carrier_* and *V_dilution_. V_dilution_* is varied so that *V_total_* is maintained at 550 sccm. The VOC concentration is defined as the flow ratio, *V_ratio_,* given by:

(1) $$V_{ratio}\left(\%\right)=\frac{V_{carrier}}{V_{carrier}+V_{dilution}}\times 100=\frac{V_{carrier}}{V_{total}}\times 100$$

The VOC concentration (*V_ratio_*) for *V_carrier_* values of 15, 30 and 60 sccm correspond to 2.73%, 5.45%, and 10.91%, respectively.

The gas mixture containing the desired VOC concentration is flowed through the gas chamber where the gas sensing event takes place. The gas mixture containing the VOC species is allowed to flow through the chamber during the ON state for 21 min. Next, the VOC molecules are purged from the chamber with purified N_2_ during the OFF state for 21 min. The transition between the ON and OFF states is automatically executed with the help of computer software. To ensure the repeatability of the sensor response, the experiment at each *V_ratio_* is repeated three times for all VOC molecules and the mean and standard deviation are computed. The standard deviation can be attributed to the variation in the temperature of the bubbler. The experiment is conducted under low RH (<15%) to ensure high sensitivity of the sensor towards VOC molecules [22] and the temperature inside the chamber is regulated at 19.5–20.5 °C with a thermostat attached to the temperature-controlled box.

### 2.3. Sensor Data Acquisition

The sensitivity response of the fabricated sensor after exposure to VOC vapors is recorded by a network analyzer (Keysight Technologies, Santa Rosa, CA USA). Resonance characteristics of the LCM sensor such as series and parallel resonance frequencies are recorded from the *admittance* (Y-mode) and *ampedance* (Z-mode) spectra, respectively. The equivalent resistance, defined as the maximum resistance, is also recorded from the impedance spectrum. The results are tabulated and analyzed with the aid of MATLAB.

## 3. Results

### 3.1. Sensing Material Characterization

#### 3.1.1. Raman Spectroscopy

**Figure 2:**
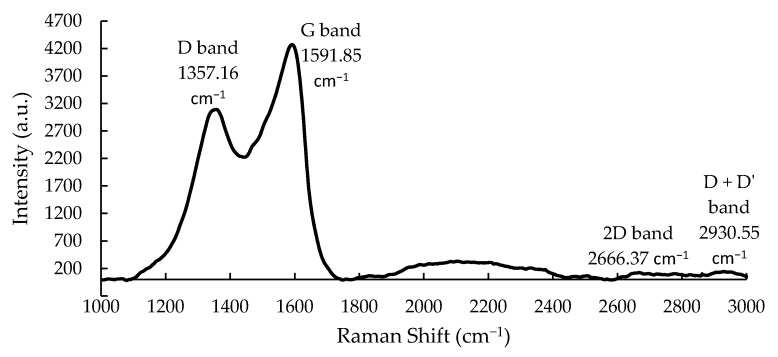
The Raman spectrum of vacuum annealed Pt-GO-LCM sample.

The in situ Raman spectrum of the Pt-GO nanocomposite on the LCM electrode is shown in Figure 2. It reveals the characteristic Raman peaks of graphene including the D band and G band at 1357 cm^−1^ and 1591 cm^−1^, respectively. The D band originates from the in-plane breathing mode of A_1g_ symmetry phonons [23]. It is activated by structural disorder in the crystal lattice due to the grain boundaries and intentional sp^3^ defects such as the functional groups through a double resonance Raman process. The G band arises from a first-order Raman process attributed to the stretching mode of E_2g_ symmetry phonons that represent the in-plane vibrations of the sp^2^ carbon atoms [23]. At higher spatial frequency, two distorted peaks which correspond to the 2D band and D + D’ band are observed at 2666 cm^−1^ and 2930 cm^−1^, respectively. The 2D band is an overtone of D band resulting from second-order Raman scattering. It is responsive towards the crystallinity and electronic band structure of graphene [23]. The D + D’ band is a combination mode that is also sensitive to the disorder in the crystal lattice [23]. The broadened 2D and D + D’ bands with relatively low intensities imply irregular stacking of multilayer graphene nanosheets and presence of intentional defects in the sample [12,23,24].

#### 3.1.2. Field-Effect Scanning Electron Microscopy (FESEM)

**Figure 3:**
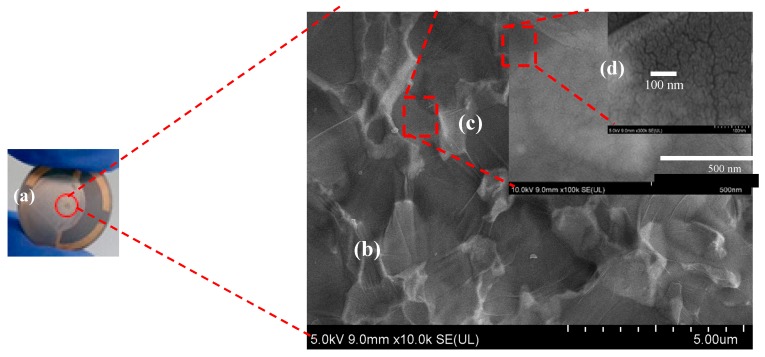
Photograph of as-fabricated Pt-GO-LCM sensor (**a**) and FESEM images obtained from the central Au electrode region of the device at 10,000× (**b**), 100,000× (**c**), and 3,000,000× (**d**) magnifications.

FESEM images were taken at three different magnifications of 10,000×, 100,000×, and 3,000,000×. Example images displayed in Figure 3b–d show the surface morphologies of GO flakes and Pt nanostructures present around the central part of the LCM, the gold electrode region that is most sensitive to surface perturbations. Figure 3a shows the as-fabricated sample with GO thin film covering the central electrode region. Figure 3b illustrates the surface morphologies of the GO nanosheets. The Pt nanostructures can be observed in Figure 3c,d as small, uniformly distributed white dots. To further confirm the presence of the Pt nanostructures, x-ray photoelectron spectroscopy of the Pt-GO-LCM samples was carried out as discussed below.

#### 3.1.3. X-ray Photoelectron Spectroscopy

**Figure 4:**
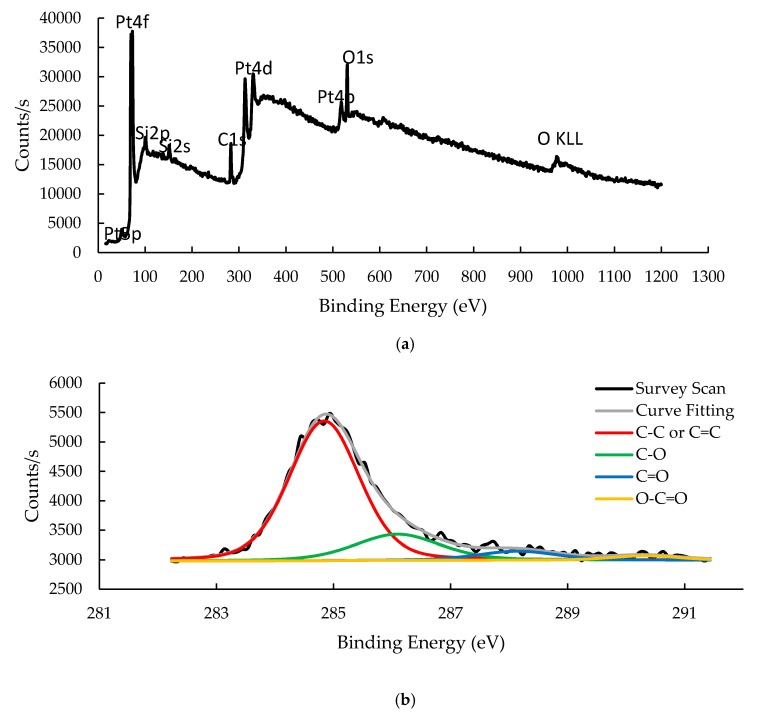

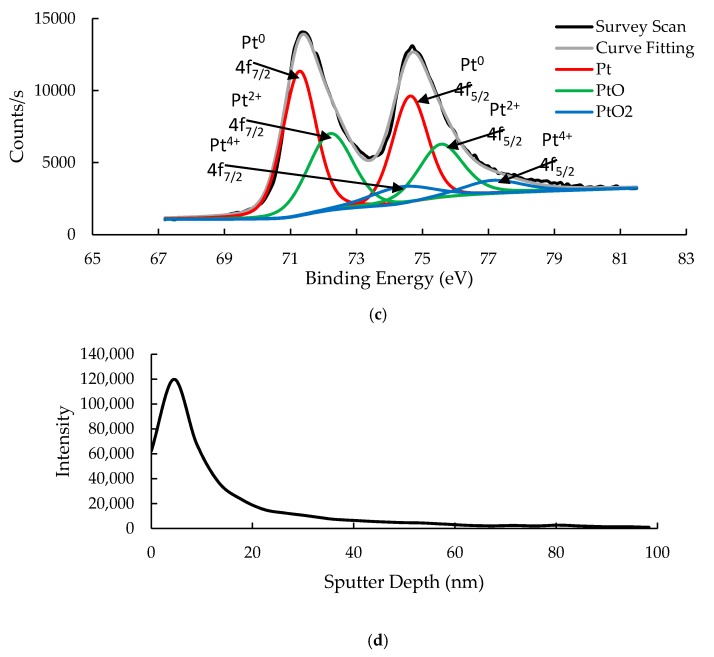
The XPS spectra of Pt-GO-LCM. (**a**) Survey scan. (**b**) Deconvolution of the C1s peak. (**c**) Deconvolution of the Pt4f region. (**d**) Depth profile of the sputtered Pt nanostructures.

XPS of the sensing material was performed to identify and quantify the surface composition together with the chemical state of the elements including carbon, oxygen, and platinum. The presence of these elements is evident from the survey scan spectrum in Figure 4a revealing three corresponding peaks denoted as C1s, O1s and Pt4f. In terms of atomic concentration, C, O, and Pt contribute 50.24%, 27.57%, and 22.20%, respectively. In addition, there are two silicon peaks (Si1s and Si2p) which are attributed to the LCM substrate. Narrow scan of the C1s and Pt4f peaks together with the associated deconvolutions are presented in Figure 4b–c. The deconvoluted spectrum in the C1s region divulge the chemical states (functional groups) of C-C and C=C (sp^2^ carbon atoms), C-O (hydroxyl, phenol, ether of tertiary alcohol, epoxy), C=O (carbonyl), and O-C=O (carboxyl) with binding energies of 284.84, 286.09, 288.15 and 290.32 eV, respectively [24,25,26]. The atomic concentrations of these chemical states are 74.54%, 17.42%, 5.64%, and 2.40%, respectively. The very high intensity of the C-C/C=C spectrum indicates that majority of the carbon atoms of graphene remains in the sp^2^ hybridization state. The existence of carbonaceous bonds with oxygen moieties confirms that some areas of the graphene are oxidized with sp^3^ hybridization. The spectral deconvolution of the Pt4f region resolves into three doublets that correspond to three distinct charge states of Pt element [27]. The most intense doublet at binding energies of 71.27 and 74.63 eV is attributed to metallic Pt^0^. A second doublet at binding energies of 72.21 and 75.56 eV is related to the oxidation state of Pt^2+^. The third doublet with binding energies of 74.19 and 77.14 eV is ascribed to the higher oxidation state of Pt^4+^. The chemical states of Pt^2+^ and Pt^4+^ indicate the formation of PtO and PtO_2_ compounds, respectively. 51.82% of the Pt atoms is in the Pt^0^ chemical state, 36.50% in Pt^2+^ chemical state, and 11.68% in Pt^4+^ chemical state. Depth measurement with XPS was also performed to determine the thickness of the Pt nanostructures. The depth profile of the sputtered Pt nanostructures is presented in Figure 4d, suggesting average thickness of 6 nm.

### 3.2. Functional Groups and the Sensing Mechanism

The presence of various oxygen containing functional groups attached to the graphene layer gives GO its p-type semiconductor characteristic [12,16]. There may also be oxygen molecules (O_2_) adsorbed on the surface of GO exposed to ambient air. These oxygen atoms draw freely-moving graphene π-electrons towards themselves. This causes the formation of ionized oxygen and depletion of electrons (accumulation of holes) in the graphene layer leading to the p-type behaviour of GO. The decoration of GO with Pt nanostructures is to further enhance the p-type characteristic and catalyze the oxidation of adsorbed VOC molecules [12]. The work function of Pt is higher than that of graphene [17]. Thus, electrons will preferentially move toward the Pt nanostructures that further reduces the electrons of graphene. The Pt nanostructures together with the presence of oxygen atoms act as the adsorption sites for the VOC molecules. When the sensor is exposed to VOC molecules, the oxidation of these molecules occurs via several complex processes as explained in the literature [12,16]. The VOC oxidation process entails the movement electrons back to the graphene layer, and thus reducing the hole concentration and increasing the equivalent resistance of the sensor. When the VOC molecules are removed during the desorption process, the electrons move back to the Pt adsorption sites. This in turn increases the hole concentration of graphene and reduces the equivalent resistance of the sensor.

### 3.3. VOC Sensing Results

The sensitivity of the Pt-GO-LCM sensor towards three distinct species of VOC molecules, namely, ethyl acetate, ethanol, and acetic acid, at varying concentrations was measured. The selected VOCs belong to ester, alcohol, and carboxylic acid groups, respectively. The sensor can output multiple response parameters upon exposure to the VOC molecules. In this section, the discussion is focused on the measurement of the electrical and mechanical perturbations on the surface of the sensor in the presence of analyte gas molecules.

#### 3.3.1. Electrical Sensitivity

**Figure 5:**
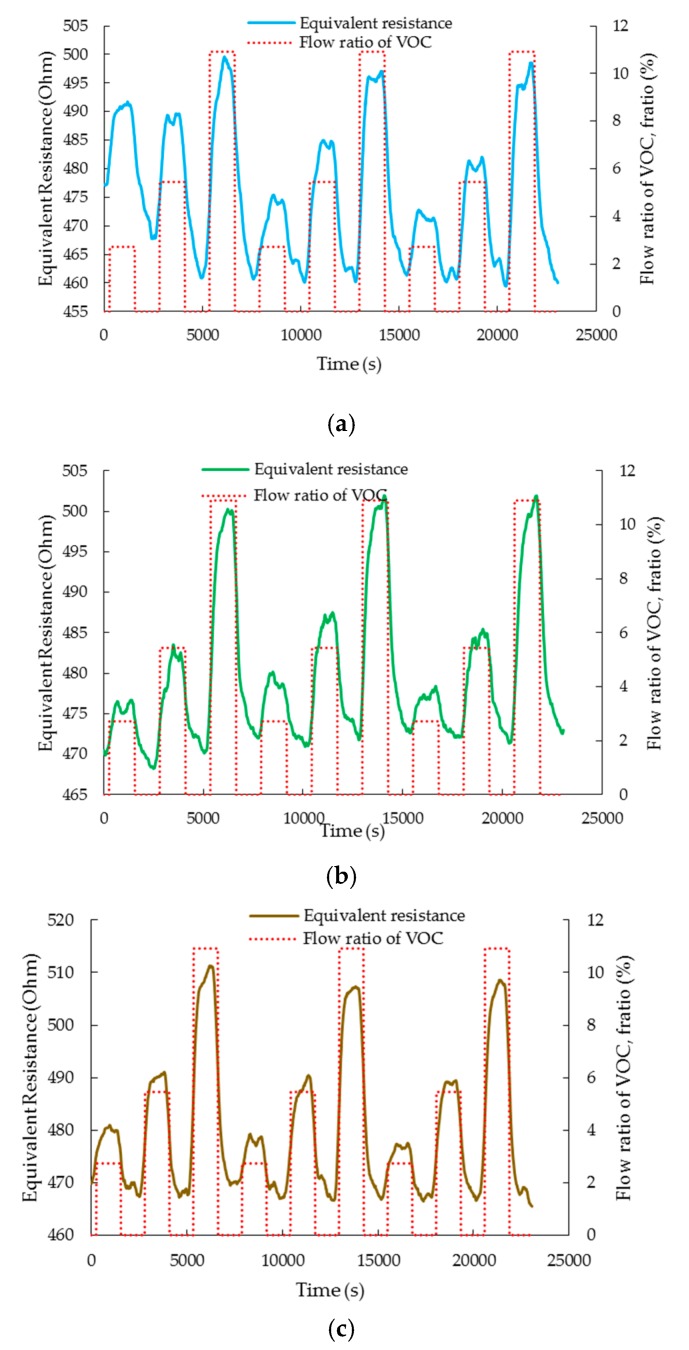
The dynamic response of the sensor’s equivalent resistance (**a**)–(**c**) towards flow ratios of 2.73%, 5.45%, and 10.91% of ethanol, acetic acid, and ethyl acetate VOC molecules, respectively. The flow ratio variation of the VOC is shown in the secondary vertical axis. A value of *V_ratio_* = 0 indicates the absence of VOC molecules in the flow.

**Figure 6:**
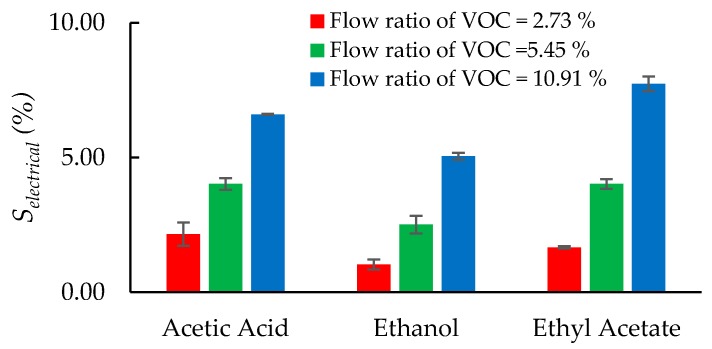
Perturbation in the equivalent resistance of the sensor upon exposure to various VOC molecules.

The electrical response of the sensor is defined as the change in the equivalent resistance of the equivalent circuit of the LCM. The electrical sensitivity, $S_{electrical},$ of the sensor is calculated as:

(2) $$S_{electrical}=\frac{R_{voc}-R_{nitrogen}}{R_{nitrogen}}\times 100,$$

where $R_{voc}$ and $R_{nitrogen}$ are the equivalent resistances of the LCM during the VOC adsorption and desorption phases, respectively. Figure 5a–c show the dynamic electrical response of the Pt-GO-LCM sensor for *V_ratio_* of 2.73%, 5.45%, and 10.91%, respectively. As observed from the plots, the equivalent resistance of the sensor rapidly increases during the ON state, when any VOC is introduced to the gas chamber. Once the resistance value saturates and reaches a steady state, the value is maintained for the duration of the ON state. After the VOC supply is cut off in the OFF state, the resistance value gradually recovers back to its original value. The trend in equivalent resistance change for each VOC follows very closely the flow ratio of the VOCs. The observed trend of increasing equivalent resistance upon exposure to VOC molecules corroborates the sensing mechanism described in the previous section. From Figure 6, it is discerned that the equivalent resistance of the sensor increases by 2.16%, 4.02%, and 6.60% upon exposure to acetic acid molecules for flow ratio of 2.73%, 5.45%, and 10.91%, respectively. When the sensor is exposed to ethanol vapors, the sensor’s equivalent resistance shifts upwards by 1.03%, 2.51%, and 5.05% for increasing order of VOC concentrations. For the same sequence of VOC concentrations, the equivalent resistance of the sensor rises by 1.67%, 4.02%, and 7.74%, respectively when ethyl acetate gas is flowing through the gas chamber. The magnitude of equivalent resistance shifts is in the increasing order of ethanol, acetic acid, and ethyl acetate which implies that the equivalent resistance shifts are contingent on the dielectric constants of the VOC analytes. Ethanol, acetic acid, and ethyl acetate have dielectric constants of 25.3, 6.2, and 6.0184, respectively [28]. Ethanol, with the largest dielectric constant, causes the smallest equivalent resistance shifts while ethyl acetate having the smallest dielectric constant effects the largest equivalent resistance shifts. Thus, the magnitude of equivalent resistance shifts for acetic acid is between that of ethanol and ethyl acetate.

#### 3.3.2. Mechanical Sensitivity

**Figure 7:**
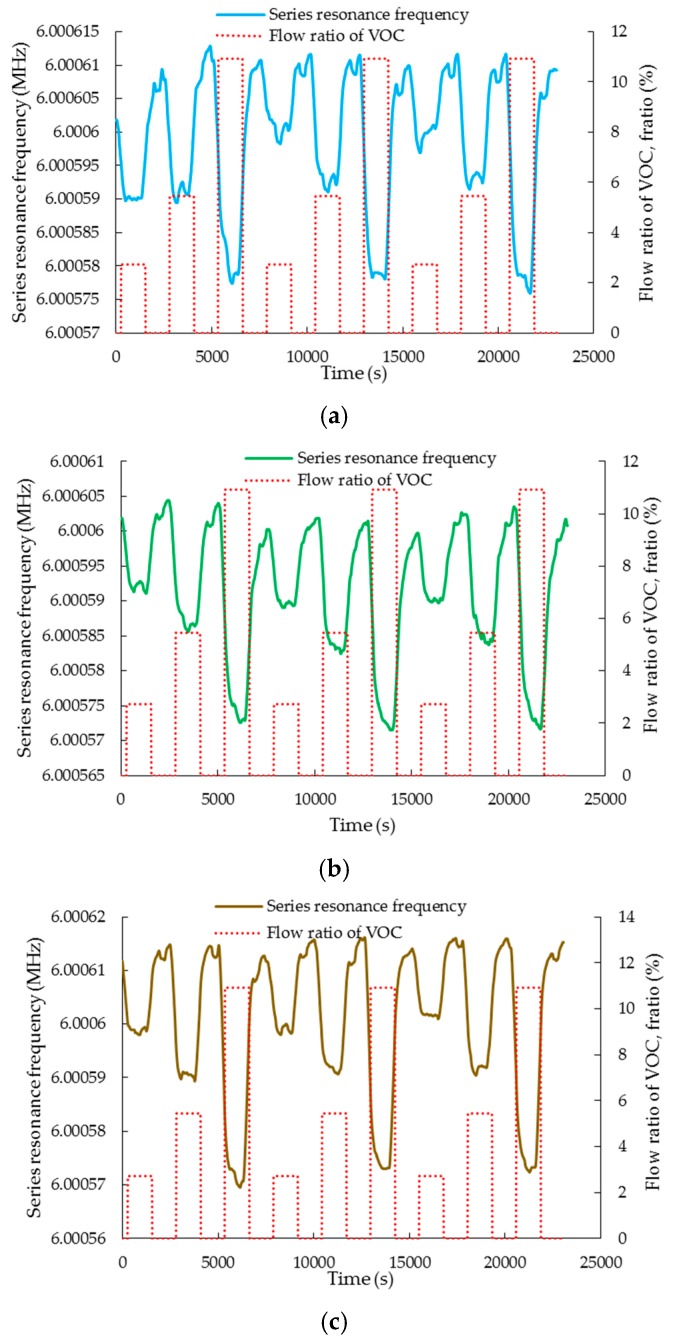
The dynamic response of the sensor’s series resonant frequency (**a**)–(**c**) towards flow ratios of 2.73%, 5.45%, and 10.91% of ethanol, acetic acid, and ethyl acetate VOC molecules, respectively. The flow ratio variation of the VOC is shown in the secondary vertical axis. A value of *V_ratio_* = 0 indicates the absence of VOC molecules in the flow.

**Figure 8:**
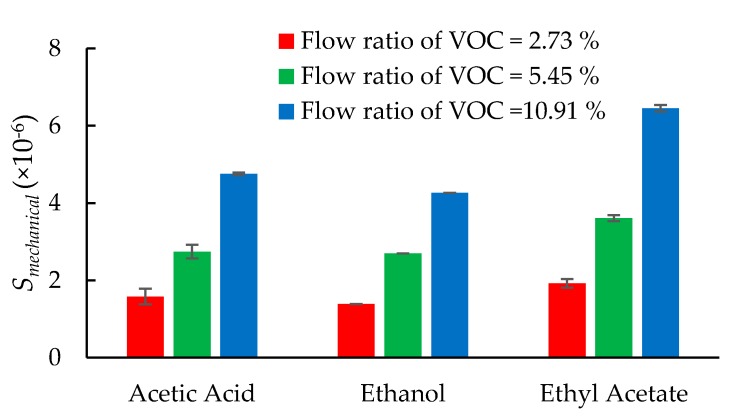
Perturbation in the series resonant frequency of the sensor upon exposure to various VOC molecules.

The mechanical perturbation (mass loading) on the sensor is measured by the series resonant frequency of the sensor. Figure 7a–c shows the dynamic series resonant frequency downshift of the sensor when it is exposed to VOC molecules. The decrement in the series resonant frequency increases in proportion to increasing *V_ratio_*. The absolute mechanical sensitivity for a change in flow ratio of the VOC, *S_mechanical_* of the sensor, is computed as below:

(3) $$S_{mechanical}=\left|\frac{f_{voc}-f_{nitrogen}}{f_{nitrogen}}\right|$$

where *f_VOC_* and *f_nitrogen_* are the series resonant frequencies of the LCM during the VOC adsorption and desorption phases, respectively. Similar to equivalent resistance shifts, the change in series resonant frequencies also increases with increasing concentrations of VOC. Figure 8 shows the plot of *S_mechanical_* obtained for different values of *V_ratio_* and VOC types. In general, it can be noted that the absolute mechanical sensitivity, *S_mechanical_,* increases with increasing flow ratios of VOCs. In other words, the series resonant frequency shows a decreasing trend with increasing mass loading caused by the flow of VOC molecules according to the well-established Sauerbrey equation [29]. It can be further observed from Figure 8 that the magnitude of *S_mechanical_* depends on the molecular weight of the VOC species [28]. Ethanol with its lowest molecular weight of 46.07 gmol^−1^ has the smallest shifts while the largest shifts are observed for ethyl acetate, which has the largest molecular weight of 88.11 gmol^−1^. The molecular weight of acetic acid is 60.05 gmol^−1^. Hence, the magnitude of series frequency shift for acetic acid is between that of ethanol and ethyl acetate.

Furthermore, it should also be noted that each flow ratio is repeated three times for all the VOC species studied to ensure that the results obtained are repeatable. The cyclic responses observed in Figure 5 and Figure 7 demonstrate high degree of repeatability for all VOC experiments in terms of both electrical and mechanical sensitivities.

#### 3.3.3. Sensitivity Comparison with Bare LCM

Compared to the bare LCM electrode surface, the GO-Pt nanostructure-coated LCM electrode surface is more conductive. This observation is corroborated by the decrease in the equivalent resistance of the Pt-GO-LCM from approximately 1.1 kΩ to about 460 Ω. It was observed that upon exposure to VOC the mechanical sensitivity of the bare LCM is almost non-existent as there is no obvious trend in the changes of the series resonant frequency and negligible changes in the equivalent resistance of bare LCM. Hence, with the addition of the thin film containing GO and Pt nanostructures as the sensing medium, both mechanical and electrical sensitivities have been enhanced.

#### 3.3.4. Selectivity

**Figure 9:**
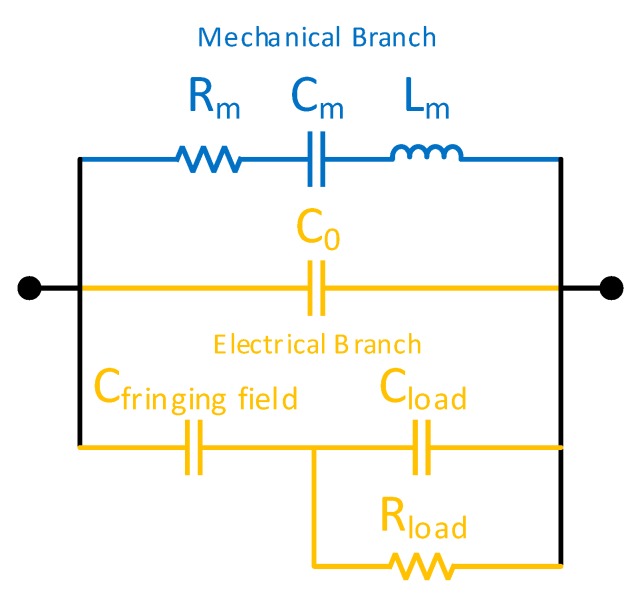
The equivalent circuit of the Pt-GO-LCM.

**Figure 10:**
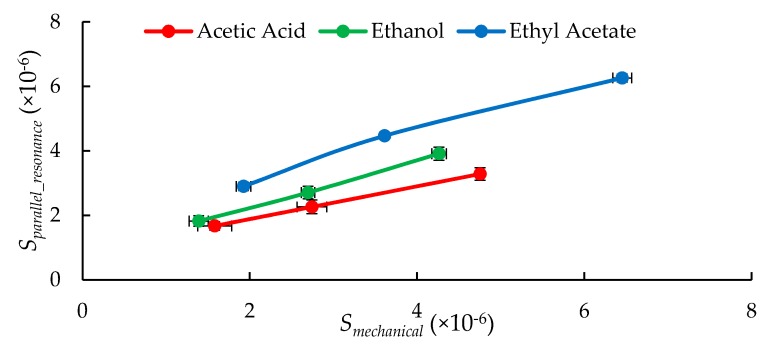
Plot of sensitivity of series versus parallel resonant frequencies for a change in flow ratio of VOC gas over the Pt-GO-LCM indicating the selectivity property of the sensor to discriminate the VOC species under study.

In addition to the two response parameters discussed in the forgoing sections, the sensor can also output another response parameter known as the parallel resonance frequency. It is the frequency when the impedance of the resonator is at maximum. Figure 9 shows equivalent circuit of proposed Pt-GO-LCM sensor. It can be noted that *R_m_*, *C_m_*, *L_m_* and *C*_0_ are typical impedance parameters of a LCM resonator equivalent circuit as described in [20]. By taking account on the perturbations, one can introduce parallel impedance elements such as the *R_load_* and *C_load_* attributed to the flow of the VOC molecules and *C_fringing field_* contributed by the electric fields that extend outside the LCM electrode region. *R_load_* represents the conductivity change while *C_load_* represents the change in dielectric constant attributed to the different VOC molecules. These parallel impedance elements are illustrated in Figure 9. Different species of VOC molecules affect these impedance elements distinctly. The sensor can achieve selectivity to discriminate the VOC molecules by using the data provided by the series and parallel resonant frequencies of the sensor. The former measures only the mass loading while the latter measures both the mass and electrical loadings on the surface of the sensor in the presence of VOC molecules. Accordingly, parallel resonance frequency of the LCM was also measured using the network analyzer. Furthermore, parallel resonance sensitivity for a change in flow ratio was estimated using Equation (4) given below:

(4) $$S_{parallel\_resonance}=\left|\frac{f_{parallel\_voc}-f_{parallel\_nitrogen}}{f_{parallel\_nitrogen}}\right|,$$

where *f_parallel_VOC_* and *f_parallel_nitrogen_* is parallel resonance frequency of the LCM recorded when VOC gases with nitrogen and nitrogen alone were flown, respectively. The *S_parallel_resonance_* versus *S_mechanical_* data presented in Figure 10 suggest that the lines representing the VOC species tested do not intersect. Thus, by knowing series and parallel frequency values together, the VOC species can be discriminated. It can be noted that the *S_parallel_resonance_* follows linear trend with *S_mechanical_*. This is because parallel resonance of LCM is contributed by both mechanical and electrical branch (see Figure 9) of LCM and are dependent on concentration of VOCs. Hence, the discrimination of VOCs is limited by the concentration of VOCs under study. However, this limitation and spacing between the lines in Figure 10 can be improved with optimized sensing medium.

## 4. Conclusions

In summary, a LCM electrode surface coated with a thin film of graphene oxide and platinum nanostructure as the sensing medium for detection and discrimination of VOC molecular species was investigated for the first time. Successful deposition of the GO–Pt nanocomposite thin film using spin-coating (for GO) and sputtering (for Pt) on the gold electrode region of the LCM was confirmed using Raman spectroscopy, FESEM, and XPS. The fabricated Pt-GO-LCM sensor was then exposed to fixed concentrations of selected VOC species in a gas chamber maintained at 20.0 ± 0.5 °C and the LCM parameters such as the series resonance frequency, parallel resonance frequency, and equivalent resistance were recorded using a network analyzer. The sensitivity values for a change in flow ratio of the VOC species were also estimated using the LCM parameters. Ethanol, acetic acid, and ethyl acetate, belonging to alcohol, carboxylic acid, and ester groups, respectively were considered as example VOCs for the study. The mass loading by the VOC molecules effected series resonance frequency changes, whilst the physical interactions with the VOC molecules altered the conductivity properties of the GO-Pt sensing film, observable as changes in equivalent resistance and parallel resonance frequency of the LCM. For a given concentration of the VOC vapors, the highest change in equivalent resistance was observed for ethyl acetate, followed by ethanol and acetic acid. Similar trend is also observed for parallel resonance frequency shift. Thus, whilst the series resonance frequency change aided in detecting the VOC concentration, the impedance changes and parallel resonance frequency shifts of the LCM aided in discriminating the VOC types. This work opens up opportunity to develop novel physical sensors capable of detecting and discriminating VOCs that can function not only at room temperature but also at elevated temperatures, limited only by the thermal stability limit of the employed sensing film. The advantage of both mechanical and electrical sensitivities of LCM enables its use not only for the detection but also for discriminating various classes and types of VOCs.
